# Learning from partnered work in the growth of family medicine training globally

**DOI:** 10.3389/fmed.2026.1870134

**Published:** 2026-07-06

**Authors:** Esther M. Johnston, Mora Claramita, Putu Aryani, Bassim Birkland, Shailendra Prasad, Klaus B. Von Pressentin

**Affiliations:** 1 Department of Family Medicine and Community Health, University of Minnesota, Minneapolis, MN, United States; 2 The Global Engagement Network for Primary Health Care (GEN-PHC), Minneapolis, MN, United States; 3 Department of Family and Community Medicine, Universitas Gadjah Mada, Yogyakarta, Indonesia; 4 Department of Public Health and Preventive Medicine, Faculty of Medicine, Udayana University, Bali, Indonesia; 5 Department of Community and Family Medicine, University of Zambia, Lusaka, Zambia; 6 Seed Global Health, Lusaka, Zambia; 7 Division of Family Medicine, Department of Family, Community and Emergency Care, University of Cape Town, Cape Town, South Africa

**Keywords:** Family Medicine and Primary Care, general practice, global health, health systems, partnership, primary health care

## Abstract

Family medicine is a foundational primary care discipline with tremendous global variation in its composition and implementation. Family medicine academic programs are similarly unique in their structure and training approaches. Across the world, family medicine training programs experience a range of resources and barriers to implementing education. Partnerships offer a unique way to share relevant expertise and navigate challenges. In this paper, we utilize the examples of family medicine programs from three different settings, at three different stages of development, to illustrate how partnerships function to fuel primary health care transformation. By analyzing these distinct stages, we highlight how partnered work evolves from foundational workforce education to system-wide integration and offer a roadmap for potential future collaborations. This study demonstrates that education models do not exist in isolation; rather, linkages between academic units and other educational partners, non-profit and professional organizations, community and governmental partners strengthen the possibility of realizing a primary health care framework to achieve health for all.

## 1 Introduction

Family medicine is a unique discipline: grounded in first contact, comprehensive, coordinated and continuous care, situating the individual patient in the context of community and population health, it is often thought of as the primary care medical specialty (1). Data suggests that the impact of a specialist trained in generalism is profound: family doctors provide improved care of chronic conditions even compared to disease-specific specialist-provided care (2). A primary health care system grounded in family medicine provides high-quality care for the greatest number of people at the lowest cost (3). Yet family medicine as a discipline is still emerging - it first developed as a formal medical specialty in the 1950s and 1960s across the UK, Canada, and the United States, and has since grown into a worldwide phenomenon. In some countries, health systems are still in an exploratory phase, understanding how family medicine might be integrated, and in others, the first training programs are now being developed (4).

The scope and practice setting of a family doctor differ dramatically across regions and local contexts. Family medicine training varies in length across the globe, as do the size of family medicine academic units, and even the settings in which those units exist (4). In some areas, family medicine training is housed inside formal academic environments, while in others it may be hosted directly by community health centers or hospitals. This variation in design is a direct response to the imperative to produce graduates capable of meeting their communities’ needs.

Across the world, family medicine training programs have varied resources and barriers to implementing education. They collaborate with training centers, non-profits, professional organizations, and governments to share expertise, resources, and address challenges. A previous study of family medicine academic units highlighted the opportunity for such collaborations to strengthen faculty development, research, and joint advocacy (5). Academic units may seek and achieve different partnerships depending on their stage of program development. Accordingly, they may have different resources to offer to a partner as their program develops.

This exemplar study examines family medicine education across three settings at various stages of program development to illustrate how the needs and structures of collaborations and partnerships change over time. We chose this methodology as an approach to identifying and studying entities that represent ideal cases or outliers of a specific concept or construct (6). We highlight how partnered work evolves from foundational workforce education to system-wide integration and offer a roadmap for potential future collaborations.

## 2 Stages of program development

To provide context for our examples, we classify family medicine programs as follows:

- Early-stage programs: lasting from the program’s conception through graduation of its second class. At this stage, the program is primarily focused on laying its foundations and identifying how it must adapt to simultaneously meet the requirements of sponsoring educational institutions and professional/licensing bodies, as well as the community’s needs.
- Intermediate-stage program: beginning with the graduation of the program’s second class and may focus on mentoring existing faculty and developing new faculty.
- Established programs: established programs have produced 10 or more years of graduates. Some graduates have become faculty and mentored to mid-stage career in this role. Some faculty are now “late career.”

Using purposive sampling, we identified three exemplars, from Indonesia, Zambia, and South Africa, to illustrate the strengths and needs of partnerships by programs at each of the above stages.

## 3 Indonesia

Indonesia is a Southeast Asian country with unique geographical challenges to achieving universal primary health care: it is the largest archipelago country, and the fourth largest country by population size in the world (7). Indonesia faces a triple burden of chronic illnesses, infectious diseases, and injuries (8). To meet the unique needs of its population, Indonesia implemented a national insurance system called Badan Penyelenggara Jaminan Sosial (BPJS) in 2014 (9, 10). This system utilized over one-hundred thousand general practitioners (GPs) as gatekeepers to the health system. Unfortunately, none of these GPs received postgraduate training in family medicine (11).

Although an association of family medicine has existed in Indonesia since the 1980s, Indonesia did not recognize family medicine as a specialty until 2019 (12). Legislation enabled the opening of three study programs in 2022 (Universitas Gadjah Mada - Yogyakarta, Universitas Indonesia - Jakarta, and Universitas Padjajaran - Bandung) (13).

Existing GPs resisted the early stages of family medicine’s development; partnering with them was critical to ensuring the discipline’s success. Onset of the COVID-19 pandemic was pivotal in facilitating discussions that led to the development of a formal upscaling program, where GPs tested into family medicine certification, approved by the Ministry of Health and the Medical Council. Across Sumatra, Java, Bali, and Sulawesi, 500 GPs have now been upscaled to Family Medicine specialist designation. These upscaled family medicine specialists serve as clinical preceptors for the family medicine resident trainees at community centers/Puskesmas across Indonesia.

Family Medicine residency training programs in Indonesia are in an early stage of development. A few schools have now graduated their first residents, and more training programs are in the process of opening. In total, 17 universities have launched family medicine postgraduate specialist training programs.

To facilitate partnerships to guide the growth of the discipline, the Ministry of Education in Indonesia matched universities, in which an established training program (ex: Universitas Gadjah Mada) would nurture newer programs (ex: Universitas Udayana - Bali). Established programs in these partnerships provide technical assistance, workshops, learning modules, curriculum, assessment, and quality assurance guides. They also facilitate direct mentorship and advocacy support.

Formal training is also offered through the Indonesian Society of Teachers in Family Medicine (ISTFM). In collaboration with the College and Association of Family Medicine and Primary Care, the ISTFM conducts training sessions on clinical medicine, family medicine theories and practices, curriculum development, assessment, and quality assurance development, to guide each school toward accreditation and recognition. A total of 75 teachers from 11 schools participated in one 90-h, fully online course, aimed at enabling them to provide clinical supervision, conduct research and community service, and enhance holistic, comprehensive consultation services. A separate online certification training on outcomes-based education and assessment in 2026 was attended by 52 directors and 100 lecturers from 17 medical schools (14).

The national professional association also facilitates mentorship between academicians in different program units. It has been a place where members share experiences and ideas around effective advocacy, to counter opposition to family medicine development.

Many seminars and conferences, as well as coaching, mentorship, and guest lectures for national family medicine leaders, have been designed and implemented with the support of international partners, such as the University of Minnesota and University of Edinburgh.

Thanks in large part to the support provided by academic programs to one another, facilitated by the government and national professional associations, and with support from international university partners, Indonesia now has 38 board-certified specialists in family medicine.

## 4 Zambia

The University of Zambia Family Medicine training program in Lusaka, Zambia, began formally in 2019 after several years of preparation. It aims to train community-oriented family physicians capable of strengthening primary health care delivery across Zambia, particularly at the district level (15). Zambia’s health system is highly centralized, and access to first-line care is often delivered by clinicians with limited postgraduate training. From the beginning, the vision of the 4-years Master of Medicine (MMed) in Family Medicine program has been closely aligned with Zambia national health priorities to decentralize care, improve access, and build a more resilient and equitable health system (16).

Given inadequate numbers of locally trained faculty, and limited exposure to family medicine as a specialty in Zambia, partnerships, both formal and informal, were critical to establishing the foundations of the program. Early collaborations were diverse in nature, and focused on start-up needs, namely: curriculum development, scholarship support, faculty support, medical diagnostic equipment for teaching, and early national advocacy.

As the program evolved toward the intermediate stage, with growing numbers of students, faculty and graduates, these partnerships evolved as well. Partnerships that had been focused on establishing the program transitioned out or evolved to support its next phase with an emphasis on formal establishment into the Zambian health system. Most important to this was increased engagement with the Government of Zambia at various levels.

Collaboration with the Zambian Ministry of Health and government health and education regulatory bodies ensured that the program remained aligned with workforce strategies, especially around primary health care. These partnerships and collaborations were further strengthened at the provincial, district, and facility levels, where expanding training sites into district hospitals and community-based settings brought training closer to the populations served. The deliberate situating of the training program outside tertiary hospitals, closer to the realities of community health needs, was a unique approach to training postgraduate students in Zambia. Through the selection of training sites, adapting teaching to local health priorities and emerging threats, and integrating residents into existing care teams, the program remained grounded in community engagement.

The Zambia training program, as an intermediate stage program, found that non-governmental partnerships needed to be focused and specialized. Such partnerships included faculty development, in-person and remote mentorship approaches, and exposure to regional and global developments in family medicine education.

Partnerships also supported the development of research skills, engaging both residents and faculty in contextually relevant research to inform key policy and advocacy efforts in family medicine, transitioning the program from a purely educational initiative to a contributor to the academic discourse surrounding primary health care. Partnerships also played a key role in strengthening family medicine’s professional identity in Zambia. Collaboration through regional and global bodies, such as WONCA Africa and the PRIMAFAMED network, provided a place for knowledge exchange, peer learning, and alignment with regional movements in family medicine education.

The outcomes of these partnerships have been substantial. The program has grown rapidly from its initial two residents to over 35 trainees. There are now three classes of graduates. Several alumni have remained engaged as faculty and family medicine teachers. While ongoing challenges remain, family medicine has gained formal recognition within national policy frameworks, and the program now contributes increasingly to the growth of the specialty in the region.

## 5 South Africa

Over the past three decades, the University of Cape Town (UCT) family medicine training program has developed within a complex national health system. The post-Apartheid government embraced primary health care (PHC). After family medicine was recognized as a specialty in 2007 by the Health Professions Council of South Africa (HPCSA), a 4-years MMed program was established, and trainees were appointed in newly created registrar posts to align the training of family physicians with that of other specialist disciplines (17).

Postgraduate training in the Western Cape province of South Africa began as a partnership between Western Cape Government Health (WCGH), UCT, and Stellenbosch University. Following early role ambiguity, advocacy successfully increased posts in line with South African Academy of Family Physicians (SAAFP) directives (18, 19). Today, UCT has further expanded into rural areas using decentralized training models. Joint WCGH-university appointments bridge clinical service and teaching, fostering research and program relevance (20–22).

Within UCT itself, a defining characteristic of family medicine is its commitment to a continuum of health professions education. The program contributes to undergraduate medical student teaching, medical internship, and postgraduate diploma programs (for medical officers and clinical nurse practitioners), the MMed specialist pathway, and doctoral (Ph.D.) training. Learners throughout this continuum train within overlapping service environments, reinforcing the connection between education, practice, and scholarship. The composition of the UCT faculty and leadership reflects the philosophy of “growing one’s own timber” –a concept emphasized by former Dean Prof. Bongani Mayosi, referring to the intentional development and retention of graduates as future educators and institutional leaders, consistent with his broader commitment to academic development and transformation. It now includes graduates from the UCT programmes, many of whom hold key academic and leadership positions.

The sustainability of the UCT postgraduate program depends on active engagement with national professional and academic bodies, particularly during periods of inconsistent national policy support for family medicine. Partnership with the SAAFP fosters professional identity formation, advocacy, and continuing professional development, and strengthens the connection between academic family medicine and clinical practice. These structures facilitate successful collaboration among South African universities on curriculum harmonization, establishment of training standards, and development of assessment approaches, including entrustable professional activities (EPAs), to strengthen workplace-based assessment (23). Early adoption of EPAs by the discipline of family medicine paved the way for other specialties to adopt this approach within the Colleges of Medicine of South Africa (CMSA) (24, 25). Partnership with the CMSA and its College of Family Physicians anchors postgraduate training within recognized specialist certification pathways. The Fellowship qualification became the national standard in the mid-2010’s. All registrars from the nine accredited training institutions are now required to obtain this qualification to register as a specialist family physician with the Health Professions Council of South Africa. Recent research shows that only a third of graduates are retained as specialist family physicians in public sector posts, and highlights the need to continue advocacy efforts within the national and provincial departments of health (26).

University of Cape Town also engages in regional and global family medicine networks, including the World Organization of Family Doctors (WONCA). Through these networks, UCT contributes to advocacy campaigns, policy discussions, and professional development workshops worldwide. Activities enhance training opportunities and policy input at both academic and professional levels.

South-South collaboration through PRIMAFAMED has led to improvements in district-based training programs, faculty development workshops, and curriculum adaptation initiatives across African contexts (27). Engagement with The Network: Toward Unity for Health (TUFH) and the African Health Collaborative has enabled the program to participate in global discussions and initiatives focused on social accountability and community-engaged education (28). These networks may help mitigate workforce attrition by strengthening regional professional identity and academic collaboration within the African region. They do this by creating pathways for family physicians to remain professionally engaged, connected, and supported through South–South collaboration.

Complementary South–North partnerships, such as ongoing collaborations with the University of Minnesota, have also resulted in significant achievements in faculty development, research, and the facilitation of scholarly exchanges.

The UCT postgraduate family medicine program reflects a partnership landscape that includes health services, universities, professional and academic bodies, student-led organizations, and networks. Supporting a continuum of education across service, scholarship, and community, the program demonstrates how family medicine training can be maintained through long-term partnership.

## 6 Discussion

**FIGURE 1:**
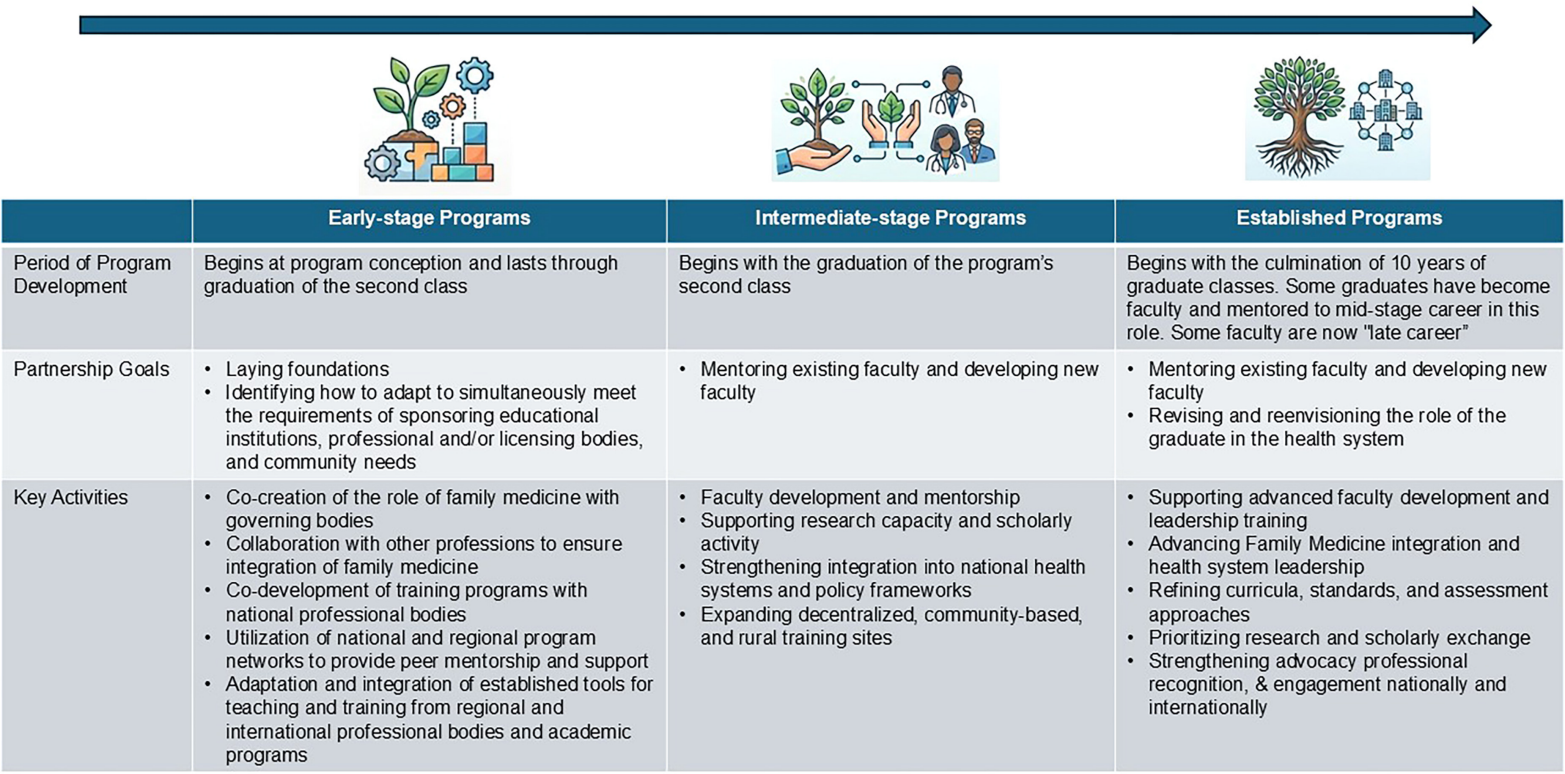
The role of partnerships at various stages of family medicine training program development.

Family medicine is an essential discipline to drive primary health care transformation. The family medicine training programs we have presented, located in different settings and at different stages of development, illustrate how collaborations by and between academic units facilitate primary health care transformation and the growth of the discipline (Figure 1).

In the initial phases, academic programs primarily focus on laying foundations. Partnerships are primarily focused on identifying the skills and competencies graduates will need to contribute to the larger health system, and the time- and resource-intensive process of building an educational program to produce those graduates. The example of Indonesia demonstrates how national partnerships with other academic programs and professional associations can be particularly impactful at this time for burgeoning programs seeking to develop their training to meet national primary health care workforce needs.

As programs progress to an intermediate stage, the focus shifts to sustainability. Programs become focused on how to recruit and retain some graduates as faculty, and the structure and content of partnerships often shift to increasingly invest in the academic department structures that sustain what was implemented in the initial phase.

As family medicine training programs become well-established, with more than a decade of graduates to represent them, the focus shifts to the role of the program in advanced systems integration and growth. As a result, partnered work matures into more complex regional and international, North–South and South–South networks that accelerate global progress toward strengthening primary health care. As the example of UCT demonstrates, these collaborations often emphasize community-engaged approaches and innovations in pedagogy to ensure the workforce remains responsive to the populations they serve. At this stage, programs may be actively and regularly generating data to support the growth of the discipline, and research partnerships become particularly valuable.

Given the changing nature of health systems and educational needs, ongoing strategic and sustainability-oriented discussions and planning within academic programs at all stages should focus on stabilizing and expanding academic capacity, strengthening supervision and research productivity, and ensuring alignment between teaching, service platforms, and applied research. The interplay between the program-specific developments and the network of partners will allow for ongoing opportunities to remain in step with the forces of change at play.

## 7 Limitations and future directions

In this perspectives piece we offer exemplars of settings in which family medicine training units have engaged in partnership at what we consider to be three typical stages of program development. Exemplars studies may serve as a starting point to further build and enhance conceptual models. The small scale of an exemplar study offers the opportunity for bias, with its limited range of perspectives, and as such offer frameworks for future work. We anticipate that future work with a wider global range of exemplars (outside of the Sub-Saharan African and South Asian regions) could be helpful to show how our model of grading programs and the roles of partnerships could generalize and translate our learning into different contexts. Program evaluations may also be helpful in reflecting on whether certain partnerships were in fact unhelpful or even harmful in growing programs at different stages of development.

Additionally, this manuscript outlines the developmental stages of programs and does not explicitly address how institutions navigate the uneven distribution of financial, administrative, and political leverage often held by high-income country partners and external funders. To ensure genuine equity and local ownership, future studies of partnerships evaluations must critically examine the mechanisms through which local programs actively protect their academic and operational autonomy–such as maintaining independent curricular alignment with national health priorities and resisting funder-driven agendas that do not serve regional workforce needs. Incorporating this socio-political layer into the grading model would provide a more realistic and comprehensive roadmap for fostering truly balanced, mutually accountable global health partnerships.

## 8 Conclusion

At different stages of program growth, academic units may be thought of as having different core focuses: from establishing foundations to ensuring sustainability to developing complex systems integration and driving the further growth of the discipline. To be successful, partnerships with academic units must recognize and respect this change in focus and may need to transform to meet the ongoing strengths and needs of programs at different stages of their life cycle. By analyzing these distinct stages, the study highlights how partnered work evolves from foundational workforce education to sophisticated system-wide integration.

## Data Availability

The original contributions presented in the study are included in the article, further inquiries can be directed to the corresponding author.
